# Biochanin a Enhances the Defense Against *Salmonella enterica* Infection Through AMPK/ULK1/mTOR-Mediated Autophagy and Extracellular Traps and Reversing SPI-1-Dependent Macrophage (MΦ) M2 Polarization

**DOI:** 10.3389/fcimb.2018.00318

**Published:** 2018-09-11

**Authors:** Xingchen Zhao, Xudong Tang, Na Guo, Yanan An, Xiangrong Chen, Ce Shi, Chao Wang, Yan Li, Shulin Li, Hongyue Xu, Mingyuan Liu, Yang Wang, Lu Yu

**Affiliations:** ^1^Key Laboratory for Zoonosis Research, Department of Infectious Diseases, First Hospital of Jilin University, Ministry of Education, College of Veterinary Medicine, College of Food Science and Engineering, Institute of Zoonosis, Jilin University, Changchun, China; ^2^Department of Food Quality and Safety, College of Food Science and Engineering, Tonghua Normal University, Tonghua, China; ^3^Key Lab for New Drug Research of TCM, Research Institute of Tsinghua University in Shenzhen, Shenzhen, China; ^4^Jiangsu Co-innovation Center for Prevention and Control of Important Animal Infectious Diseases and Zoonoses, Yangzhou, China

**Keywords:** reactive oxygen species, autophagy, extracellular traps, polarization, *Salmonella*

## Abstract

A novel treatment regimen for bacterial infections is the pharmacological enhancement of the host's immune defenses. We demonstrated that biochanin A (BCA), an isoflavone constituent in some plants, could enhance both intra- and extracellular bactericidal activity of host cells. First, BCA could induce a complete autophagic response in nonphagocytic cells (HeLa) or macrophages (MΦ) via the AMPK/ULK1/mTOR pathway and Beclin-1-dependent manner, and BCA enhanced the killing of invading *Salmonella* by autophagy through reinforcing ubiquitinated adapter protein (LRSAM1, NDP52 and p62)-mediated recognition of intracellular bacteria and through the formation of autophagolysosomes. Second, we demonstrated that BCA could enhance the release of MΦ extracellular traps (METs) to remove extracellular *Salmonella* also via the AMPK/ULK1/mTOR pathway, not through reactive oxygen species (ROS) pathway. Furtherly, in a *Salmonella*-infected mouse model, BCA treatment increased intra- and extracellular bactericidal activity through the strengthening autophagy and MET production, respectively, in peritoneal MΦ, liver and spleen tissue. Additionally, our findings showed that BCA downregulated SPI-1 (*Salmonella* pathogenicity island 1) expression during *Salmonella* infection *in vitro* and *in vivo* to reverse the MΦ M2 polarization, which was different from the MΦ M1 phenotype caused by most of bacteria infection. Together, these findings suggest that BCA has an immunomodulatory effect on *Salmonella*-infected host cells and enhances their bactericidal activity *in vitro* and *in vivo* through autophagy, extracellular traps and regulation of MΦ polarization.

## Introduction

The wide spread of drug-resistant bacteria and the slow development of new antimicrobial agents has created a desperate scarcity of new therapeutic approaches of bacterial infections (Viveiros et al., 2012). The enhancement of the host's immune defenses through pharmacological treatment is a novel area of study (Ankomah and Levin, 2014). There is an urgent need to discover agents that can enhance both extra- and intracellular bactericidal action in the host (Viveiros et al., 2012).

The innate immune system is the first line of defense against invading microorganisms through immune response elements such as reactive oxygen species (ROS), autophagy and extracellular traps (ETs) (Medzhitov, 2010). Autophagy is the natural, regulated mechanism of the cell, in which cytoplasmic components are delivered to lysosomes and autophagosomes for degradation (Yuan et al., 2012). The classical autophagy mechanism depends on two ubiquitin-like conjugation systems: Atg4-Atg7-Atg8 or Atg7-Atg12-Atg5. These two systems are important for the formation of an early complex containing class III phosphoinositide 3-kinase, which then forms the autophagosome (Yuan et al., 2012). Recent reports have demonstrated that AMP-activated protein kinase (AMPK) and mTOR coordinate autophagy initiation in mammalian cells (Shang and Wang, 2011). ETs were recognized recently (Brinkmann et al., 2004) and can be produced by many innate effector cells including macrophages (MΦ), eosinophils, mast cells and neutrophils. ETs are fiber-like extracellular structures that can defend against infections by trapping extracellular bacteria or fungi (Brinkmann et al., 2004). NADPH oxidase (NOX2)-dependent or NOX2-independent oxidative bursts have been reported to mediate ET formation (Remijsen et al., 2011).

According to the tissue microenvironment, peripheral monocytes could polarize to different MΦs subtypes, including M1 MΦs and M2 MΦs. M1 MΦs are the classical activated MΦs, which produce pro-inflammatory cytokines such as TNF-Φ and IL-12 and clear pathogens but also cause tissue damage. In contrast, M2 MΦs are alternatively activated, which generate anti-inflammatory mediators such as IL-10, suppress facilitate wound healing and inflammation (Erbel et al., 2013). It was proved that some medicine and compounds could induce MΦ polarization to M1-like MΦs or M2-like MΦs (Gharib et al., 2014).

*Salmonella enterica Typhimurium* (*Salmonella*) is an intracellular bacterial pathogen that is the leading cause of enteric disease in humans and in animal hosts. *Salmonella* can invade, survive and replicate in phagocytic and non-phagocytic cells (Davidson et al., 2015). The results proved that *Salmonella* could escape from *Salmonella*-containing vacuoles (SCVs) and enter the epithelial cells' cytosol. They are then selected for autophagy mediated by the accumulation of ubiquitin around target bacteria and for binding of the ubiquitin recognizing adaptor proteins NDP52, P62, and LRSAM1, which is essential because the E3 ubiquitin ligase is responsible for antibacterial ubiquitin association (Thurston et al., 2009; Cemma et al., 2011). Especially, it has been reported that *Salmonella* infection can polarize MΦs toward M2 phenotype that was dependent on the expression of *Salmonella* pathogenicity island 1 (SPI-1) (Kyrova et al., 2012), the M2 polarization caused by *Salmonella* infection was different from other bacteria infection that caused MΦ M1 phenotype polarization.

Until recently, few agents were found to enhance bacterial clearance by promoting anti-bacterial autophagy (Conway et al., 2013) or ETs (Chow et al., 2010). In this study, we found that Biochanin A (BCA), a major isoflavone constituent found in red clover, cabbage, alfalfa and some other herbal dietary supplements, could enhance bactericidal intra- and extracellular activity. BCA also has putative benefits in dietary cancer prophylaxis (Medjakovic and Jungbauer, 2008). BCA has potential antimicrobial activity against several types of bacteria, but these cases include only high minimum inhibitory concentration (MIC) values (Liu et al., 2011). BCA is a common product extracted from natural plant and is considered to be innocuous (Sklenickova et al., 2010). We found that BCA induced the autophagic response in epithelial cells and MΦs or induced ET formation of MΦs *in vitro* and in an *in vivo* mouse model. We also investigated the influence of BCA on SPI-1-dependent MΦ polarization during bacteria infection.

## Materials and methods

### Antibodies, chemicals, plasmids, and strains

BCA was purchased from Wako Pure Chemicals, Industries, Ltd. (Chuo-ku, Osaka, Japan). The following antibodies used for western blotting were purchased from Cell Signaling Technology (Massachusetts, USA): Beclin-1 antibody (3495S), p62/SQSTM1 antibody (5114S), p-ULK1ser317 antibody (6887S), p-ULK1ser757 antibody (6888S), p-p70S6K antibody (9234P), p-4E-BP1 antibody (9451P), p-mTOR antibody (5536P), p-AMPKβ antibody (4181P), β-actin antibody (4970S) and anti-rabbit horseradish peroxidase-labeled antibody (7054S). The LC3B antibody was purchased from Sigma Chemical Company (042M4774V). All other reagents were purchased from Sigma Chemical Co. (St. Louis, MO, U.S.A.) unless otherwise stated. *Salmonella* ATCC14028 was stored at −80°C.

### Cells and cell cultures

All the cells (HeLa cells, Raw264.7 cells and THP-1 cells) were bought from ATCC (Maryland, USA) and maintained in RPMI 1640 medium with additional 10% fetal bovine serum (FBS). All the culture reagents were obtained from GIBCO Laboratories (NY, USA). The cells were incubated at 37°C with 5% CO_2_. Phorbol myristate acetate (PMA) (Sigma, Missouri, USA) with the concentration of 10 ng/ml for 24 h was used to induce the THP-1 cells to differentiate into MΦ-like cells.

### Transmission electron microscopy

The specimen preparation was performed as other research report described (Yuan et al., 2012). A transmission electron microscope (Hitachi H-7650, Tokyo, Japan) was used to observe the autophagosome-like vesicles.

### SDS/PAGE and western blotting

Western blotting and SDS/PAGE were operated as other research report described (Mizusaki et al., 2008; Yuan et al., 2012). A CanoScan LiDE 100 scanner (Canon) was used to obtain the images. The results were quantified with Image-J software.

### Quantitative real-time RT-PCR (q-RT-PCR) assays

RNA extraction was performed with RNAprep Pure Cell/Bacteria Kit (TIANGEN Biotech, Beijing, China) according to the manufacturer's instructions. cDNA was synthesized and all PCRs were performed as previous described (Mizusaki et al., 2008). The primers used in our study were as follows: for the 16S rRNA gene, forward primer TGTAGCGGTGAAATGCGTAG and reverse primer CAAGGGCACAACCTCCAAG (predicted length of the transcript, 161 bp); for hilA, forward primer CATGGCTGGTCAGTTGGAG and reverse primer CGTAATTCATCGCCTAAACG (predicted length of the transcript, 150 bp); for hilD, forward primer ACTCGAGATACCGACGCAAC and reverse primer CTTCTGGCAGGAAAGTCAGG (predicted length of the transcript, 129 bp). An ABI Prism 7500 Sequence Detection System (Applied Bio-systems) was used to perform the real-time PCR and set in the standard run mode. To collect the data of real-time PCR, we used the Rotor Gene 6000 series software 1.7. Reaction conditions were 5 m at 94°C, and 40 cycles at 94°C for 5 s and 60°C for 35 s. All real-time PCR reactions were carried out in triplicate and were repeated from three different bacterial cultures.

### Confocal microscopy and immunofluorescence staining

The cells were grown in glass-bottomed cell culture dishes. The samples were prepared as previously described (Cemma et al., 2011). An Olympus FV1000 confocal laser scanning microscope (Olympus, Tokyo, Japan) was used to catch the images with a 60 × objective lens. The images were analyzed using Fluoview ver. 1.7.3.0 (Olympus, Tokyo, Japan).

### Detection of the quantity of extracellular DNA (eDNA)

Raw264.7 cells were seeded in 24-well plates. eDNA was labeled with 20 μM Sytox Green (Invitrogen, OR, USA) for 10 min at room temperature. The ODs were measured with a spectrofluorophotometer (Infinite F200 Pro, TECAN, Switzerland) at a 480-nm excitation and a 545-nm emission wavelength (Brinkmann et al., 2004).

### MΦ bactericidal activity assays

The intracellular bacterial killing assay was performed and evaluated by a gentamicin protection assay according to a previously published method (Wiedemann et al., 2012; Wang et al., 2016). The extracellular bacterial killing assay was performed and evaluated in accordance with a previously described method (Wang et al., 2016).

### Detection of cytosolic ROS

We used dihydrorhodamine (DHR) 123 for cytosolic ROS detection, (a fluorescent indicator of cytosolic ROS). The cells were stimulated with the indicated reagents or infected with bacteria and then incubated with 1 μM DHR 123 for 20 min without light. The results were subsequently obtained using a spectrofluorophotometer (Infinite F200 Pro, TECAN, Switzerland) at a 485-nm excitation and a 535-nm emission wavelength (Shen et al., 2016).

### Flow cytometry

Staining of cells and analysis on a flow cytometer (FACScan; BD Biosciences) were done as described (Wang et al., 2017). Briefly, M1 and M2 MΦs were identified using the following antibodies: FITC Anti-CCR7 (eBioscience, 11-1979-42), PE Anti-CD163 (eBioscience, 12-1639-41), FITC Anti-CD206 (Invitrogen, MA5-16870) and PE anti-CD86 (Bioss, bs-1035R). The data acquired were analyzed with FlowJo (Treestar software, Ashland, OR, USA).

### Quantification of cytokines

The cytokines TNF-α and IL-10 were quantified using ELISA with the Cytokine Duo-Set Kit (R&D Systems, Minneapolis, USA). The experiment was performed according the kit introduction.

### *In vivo* studies

Female BALB/c mice (8–10 weeks old) were bought from the Shanghai Experimental Animal Center (Shanghai, China). The mice were kept under pathogen-free condition before infection with *Salmonella*. The mice were infected by intragastric administration of an overnight culture of *Salmonella* (10^5^ bacteria in 0.1 ml PBS) through a gavage tube. To make sure the number of bacteria inoculated, the same overnight culture was plated onto LB agar. At 24 h postinfection, mice were assigned to treatment groups and began receiving vehicle (0.5% methylcellulose-0.1% Tween 80 in sterile water) or BCA administered intragastrically by gavage once daily (Chiu et al., 2009).

The mice were treated with vehicle or BCA at 3.125–12.5 mg/kg intragastrically and/or 3-MA at 24 mg/kg/day intraperitoneally or 1000 U DNase I intraperitoneally once daily for 4 days (10 mice for each group). The mice were sacrificed by cervical dislocation at 5 days p.i. to collect the peritoneal fluid, liver and spleen for RNA extraction, bacterial burden quantification and protein extraction (Chiu et al., 2009; Croswell et al., 2009; Yipp et al., 2012).

### Ethics statement

All animal studies were conducted according to the experimental practices and standards that were approved by the Animal Welfare and Research Ethics Committee at Jilin University (No: IZ-2009-008). The mouse experiment was performed strictly according to the National Research Council Guide for Care and Use of Laboratory Animals of People's Republic of China.

### Statistical analysis

All results were expressed the as the mean ± SD. The group means were compared using one-way ANOVA, and Student's *t*-test was used to determine significance. *P* values of 0.05 or less were considered statistically significant.

## Results

### BCA induced a complete autophagy in nonphagocytic cells and phagocytic cells

**Figure 1:**
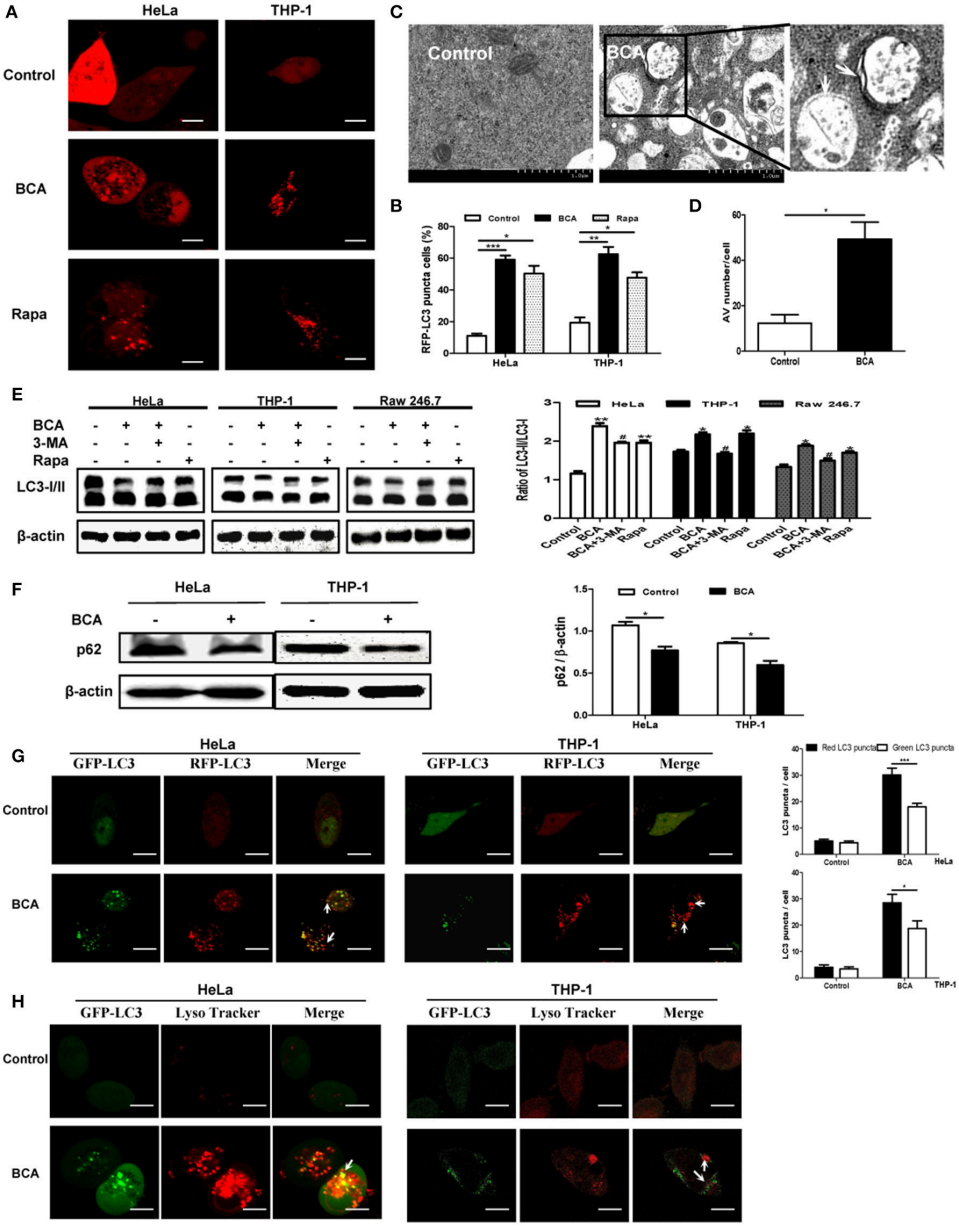
BCA induced a complete autophagy in mammalian cells. **(A,B)** HeLa and THP-1 cells were transfected with pmRFP-LC3 plasmid for 24 h and then treated with 4 μg/ml BCA for 2 h. The cells were pretreated with rapamycin (5 μM, 12 h) or 3-MA (3 μM, 3 h). Puncta in each cell were counted, and cells with more than 10 puncta were considered LC3-RFP puncta cells. The values were measured using 100 cells/sample. ^*^P < 0.05; ^**^P < 0.01; ^***^P < 0.001. **(C,D)** Treatment with BCA resulted in elevated autophagosome formation in HeLa cells. The cells were pretreated with rapamycin or 3-MA and then treated with 4 μg/ml BCA for 2 h. The cells were examined using TEM. Boxed areas are further enlarged images; arrows indicate autophagosomes. The number of autophagic vesicles (AVs) from 20 cells in each sample was determined. ^*^P < 0.05. **(E)** HeLa, THP-1 and Raw264.7 cells were pretreated with rapamycin or 3-MA and then treated with 4 μg/ml BCA for 2 h. The cells were then prepared for western blotting for LC3 was performed. The ratio of LC3-II/LC3-I was calculated. ^*^P < 0.05, ^**^P < 0.01, compared with the control group. ^#^*P* < 0.05, compared with BCA-treated cells. The data are representative of three experiments with similar results. **(F)** HeLa and THP-1 cells were treated with 4 μg/ml BCA for 2 h, and the western blotting for p62 was performed. The ratio of p62 to β-actin was calculated. ^*^P < 0.05. **(G)** HeLa and THP-1 cells were transfected with the ptfLC3 plasmid for 24 h, treated with BCA for 2 h and then observed using confocal microscopy. The arrows indicate LC3 puncta. Red or green LC3 puncta per cell were counted. ^*^P < 0.05, ^***^P < 0.001. **(H)** HeLa and THP-1 cells were transfected with pEGFP-LC3 for 24 h and treated with BCA for 2 h. The cells were incubated with 50 nM of LysoTracker for 1 h at 37°C and observed using confocal microscopy. The data are representative of three experiments with similar results.

To detect whether BCA induces an autophagic response *in vitro*, we transfected the RFP-LC3 plasmid into nonphagocytic (HeLa) and phagocytic (THP-1) cells. Our confocal microscopy results showed that more cells had LC3 puncta after BCA treatment than untreated cells (*P* < 0.001) (Figures 1A,B), which suggested that BCA could induce the autophagic response in nonphagocytic and phagocytic cells. There were significantly more autophagosome vesicles in BCA-treated HeLa cells than in control cells (*P* < 0.05) (Figures 1C,D). This demonstrated that BCA could induce autophagy. In addition, our western blot results indicated that LC3-II/LC3-I ratio increased in BCA-treated HeLa cells, Raw264.7 cells and THP-1 cells compared to their respective control cells (*P* < 0.01; *P* < 0.05; *P* < 0.05), whereas the autophagic inducer rapamycin and the autophagic inhibitor 3-MA increased and decreased the ratio of LC3-II/I, respectively (Figure 1E). These data indicated that BCA can induce the autophagic response in epithelial cells and macrophages.

Autophagy can be divided into two categories, complete and incomplete, according to whether autolysosome degradation occurs (Yuan et al., 2012). Previous studies proved that the decline of p62 expression is an important evidence for determining autophagic degradation (Klionsky et al., 2008). We found that the level of p62 expression decreased in HeLa and THP-1 cells treated with BCA (*P* < 0.05) (Figure 1F). In addition, an mRFP-GFP tandem fluorescent tagged LC3 plasmid (ptfLC3) was used in our study, which contains both red and green fluorescent proteins. When autophagosomes form, the ptfLC3 plasmid expresses both red and green fluorescence in the same puncta (Yuan et al., 2012). However, when autolysosomes form, which result from the fusion of autophagosomes and lysosomes, the GFP signal is disappeared, but RFP-LC3 is still expressed and represent LC3 puncta. In this study, after treatment with BCA, we observed the numbers of green and red LC3 puncta increased, and there were more red puncta than green puncta in BCA-treated HeLa and THP-1 cells (Figure 1G). This result confirmed the induction of autolysosome formation. In addition, we stained HeLa cells with LysoTracker DND 99, a weak lysosomotropic base that accumulates and fluoresces within acidic vesicles (Arroyo et al., 2013; Sun et al., 2014). The results showed that LC3 puncta colocalized with LysoTracker in BCA-treated HeLa and THP-1 cells (Figure 1H). This result suggested that BCA induced the acidification of autolysosomes. These data demonstrated that BCA treatment could induce a complete autophagic response and promote autophagic degradation in mammalian cells.

### BCA induced a beclin-1-dependent autophagic response mediated by the AMPK/ULK1/mTOR pathway

**Figure 2:**
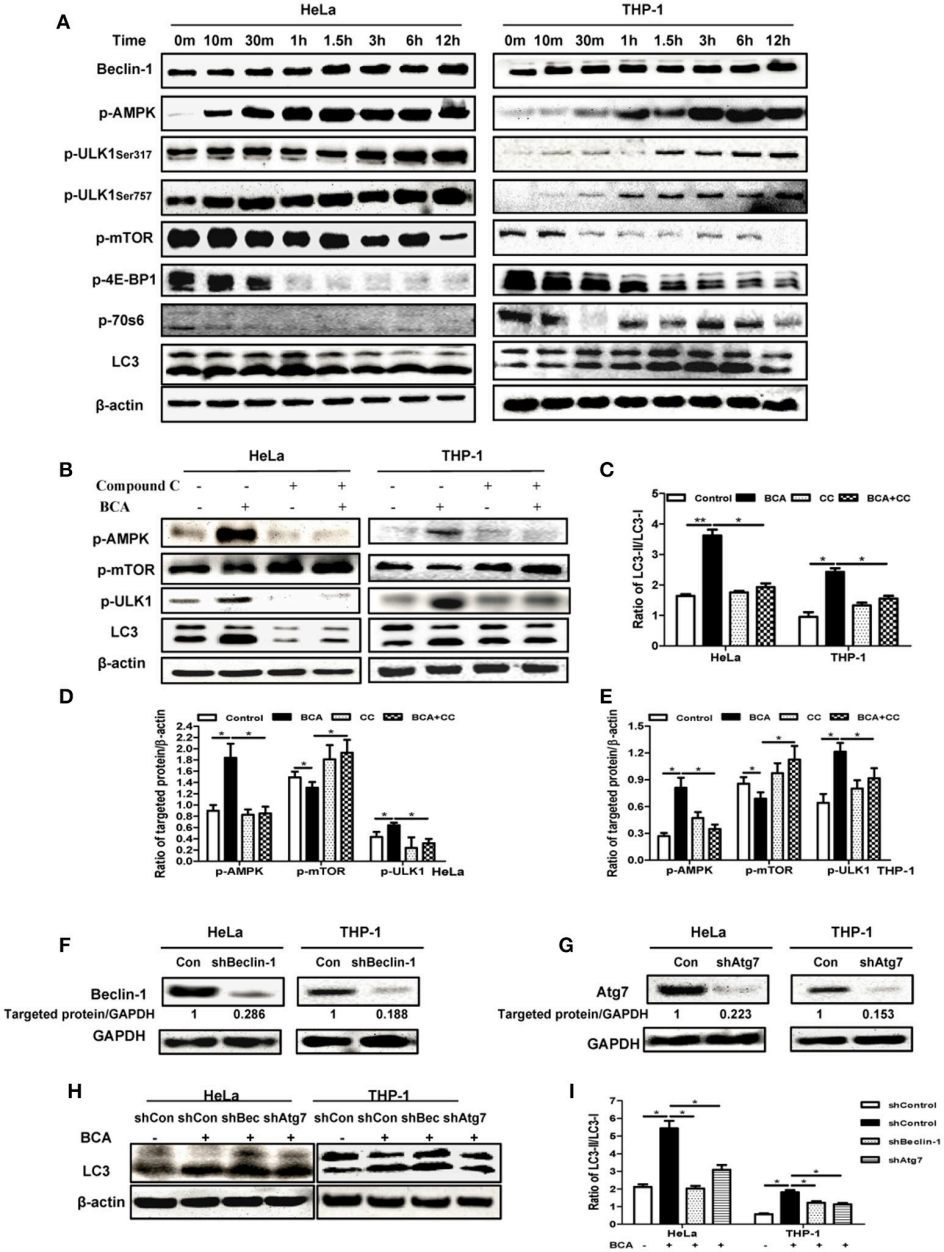
BCA induced a Beclin-1 dependent autophagic response mediated by the AMPK/ULK1/mTOR pathway. **(A)** HeLa and THP-1 cells were treated with 4 μg/ml BCA for 12 h. Then, western blotting for Beclin-1, p-AMPK, p-ULK1, p-mTOR, p-4E-BP1, p-70S6 and LC3 was performed. **(B)** HeLa and THP-1 cells were treated with 4 μg/ml BCA for 2 h with or without pretreatment with 5 μM compound C. Western blotting for p-AMPK, p-mTOR, p-ULK1 and LC3 was performed. **(C)** The ratio of LC3II/LC3-I was calculated. ^*^P < 0.05; ^**^P < 0.01. **(D,E)** The ratio of targeted protein/β-actin in HeLa or THP-1 cells was calculated. ^*^P < 0.05. **(F)** HeLa and THP-1 cells were transfected with shRNA negative control, shBeclin-1 or shAtg7 plasmids for 48 h before treatment with BCA for 2 h. **(G)** Western blotting for Beclin-1 and Atg7. **(H,I)** Western blotting for LC3 was performed. ^*^P < 0.05. The data are representative of three experiments with similar results.

In our analysis of the possible pathways involved in the BCA induced autophagic response, we found that the phosphorylation levels of p70S6K, 4E-BP1 and mTOR were obviously declined in a time-dependent manner in HeLa and THP-1 cells after BCA treatment compared to the untreated cells, while the phosphorylations of AMPK and ULK1 (Ser 317 and Ser757) were increased. The LC3-II/LC3-I ratio also increased in a time-dependent manner after BCA treatment (Figure 2A). In addition, the cells were treated with BCA and compound C (an AMPK inhibitor) to better know this pathway (Park et al., 2013). The results showed that compound C lowered AMPK and ULK1 phosphorylation and decreased LC3-II expression induced by BCA (*P* < 0.05) (Figures 2B–E), while it increased the phosphorylation of mTOR (Figures 2B,D,E). Together, these results suggested that BCA treatment activated the AMPK/mTOR/Ulk1 autophagy pathway.

In addition, we found that BCA treatment increased Beclin-1 expression in HeLa and THP-1 cells (Figure 2A), which implied that the autophagy induced by BCA might be via by Beclin-1. To further confirm the correlation between Beclin-1 and BCA-induced autophagy, we used specific shBeclin-1 plasmids to knock down Beclin-1 expression in these two cell lines (Figure 2F). The ratio of LC3-II/LC3-I in BCA-treated HeLa cells and THP-1 cells transfected with the shBeclin-1 plasmid markedly decreased compared with cells transfected with negative shRNA plasmid and control group (*P* < 0.05) (Figure 2H,I). To further investigate this pathway, we also used shAtg7 plasmids to knock down the expression of Atg7, a major autophagy related downstream protein, and the ratio of LC3-II/LC3-I in BCA-treated HeLa and THP-1 cells after Atg7-specific knockdown significantly decreased compared to the control group (*P* < 0.05) (Figures 2F–I). Taken together, these data indicated that Beclin 1 plays a crucial role in the mechanism of autophagy induced by BCA.

### BCA improved the killing of intracellular *salmonella* through autophagy by enhancing adapter protein mediated recognition of invading bacteria and the formation of autophagolysosomes

**Figure 3:**
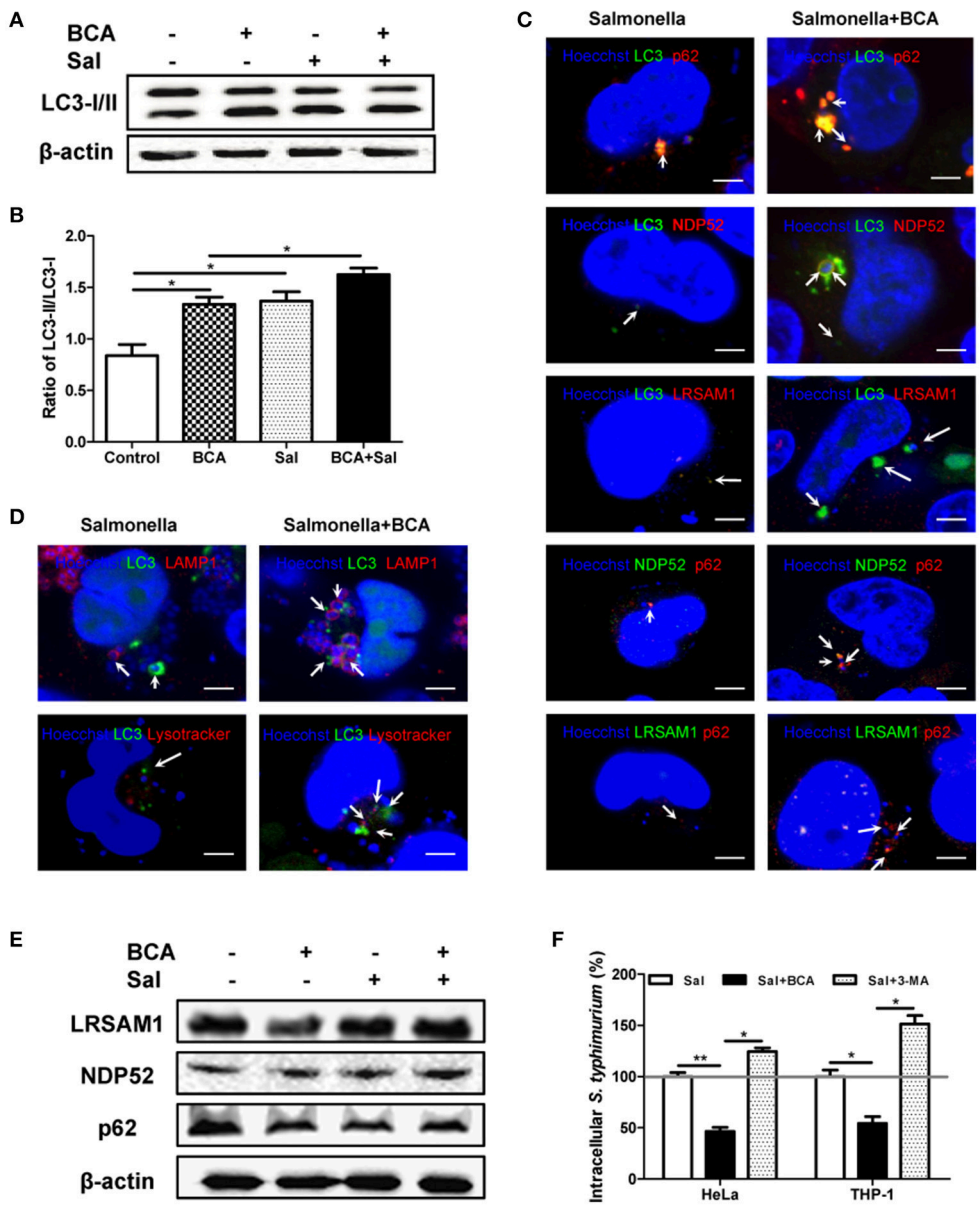
BCA enhanced the autophagic response induced by *Salmonella*. HeLa cells were pretreated with 4 μg/ml BCA for 2 h and then infected with *Salmonella* for 2 h (MOI = 10:1). **(A)** Western blotting for LC3 was conducted. **(B)** The ratio of LC3-II/LC3-I was calculated. ^*^P < 0.05. **(C,D)** The cells were immunostained with antibodies against NDP52 and p62 and Hoechst 33342 then analyzed for colocalization of the adaptor proteins and intracellular bacteria. HeLa cells were transfected with pEGFP-LC3 for 24 h before treatment with BCA and infected with *Salmonella*. The cells were immunostained with different antibodies (NDP52, p62, and LAMP1), LysoTracker and Hoechst 33342 and then analyzed for colocalization of the adaptor proteins, LAMP1, LysoTracker, LC3 puncta and intracellular bacteria and were observed using confocal microscopy. **(E)** HeLa cells were pretreated with 4 μg/ml BCA for 2 h then infected with *Salmonella* for 2 h (MOI = 10:1). Then, western blotting of LRSAM1, NDP52, and p62 was performed. **(F)** HeLa cells and THP-1 cells were treated with BCA or 3-MA and infected with *Salmonella* for 2 h, then cultured in medium containing 100 μg of gentamicin ml^−1^ for 1 h to remove the extracellular bacteria. The intracellular bacteria were counted as CFUs. ^*^P < 0.05; ^**^P < 0.01.

To examine the effect of autophagy induced by BCA on intracellular *Salmonella*, we compared LC3 expression in *Salmonella* infected HeLa cells with or without BCA treatment. *Salmonella* infection increased the LC3-II/I ratio, and pretreatment with BCA significantly enhanced the increase of the ratio (*P* < 0.05) (Figures 3A,B). Immunofluorescence staining showed that colocalization between NDP52, p62, LRSAM1, the LC3 puncta and intracellular bacteria was significantly increased in those BCA-pretreated cells (Figures 3C and Figure S1), and colocalization between LAMP1, LysoTracker, LC3 puncta and intracellular bacteria also increased significantly in those BCA-pretreated cells (Figure 3D and Figure S1). Moreover, western blot results showed that BCA enhanced the expression of ubiquitinated adapter proteins NDP52, p62, and ubiquitin ligase LRSAM1 (Figure 3E), which indicated that the enhanced expression of ubiquitinated adapter proteins NDP52, p62, and ubiquitin ligase LRSAM1 may be the direct result of restricting the proliferation of intracellular *Salmonella* through autophagy induced by BCA. A gentamicin protection assay indicated that the number of intracellular bacteria in the BCA-treated cells was significantly decreased (*P* < 0.05), whereas that of the 3-MA pre-treated cells showed the opposite result (*P* < 0.05) (Figure 3F). To investigate the anti-microbial activity on *Salmonella* alone, we also detected the minimum inhibitory concentration (MIC) of BCA and found the MIC was more than 512 μg/ml, which was much higher than the concentration we used to induce autophagy. Together, these data suggest that BCA improved the killing of intracellular *Salmonella* through autophagy by enhancing adapter proteins (LRSAM1, NDP52 and p62)-mediated recognition of invading bacteria and formation of autophagolysosome.

### BCA induced MΦ extracellular trap (MET) formation and improved extracellular bacterial killing *in vitro*

**Figure 4:**
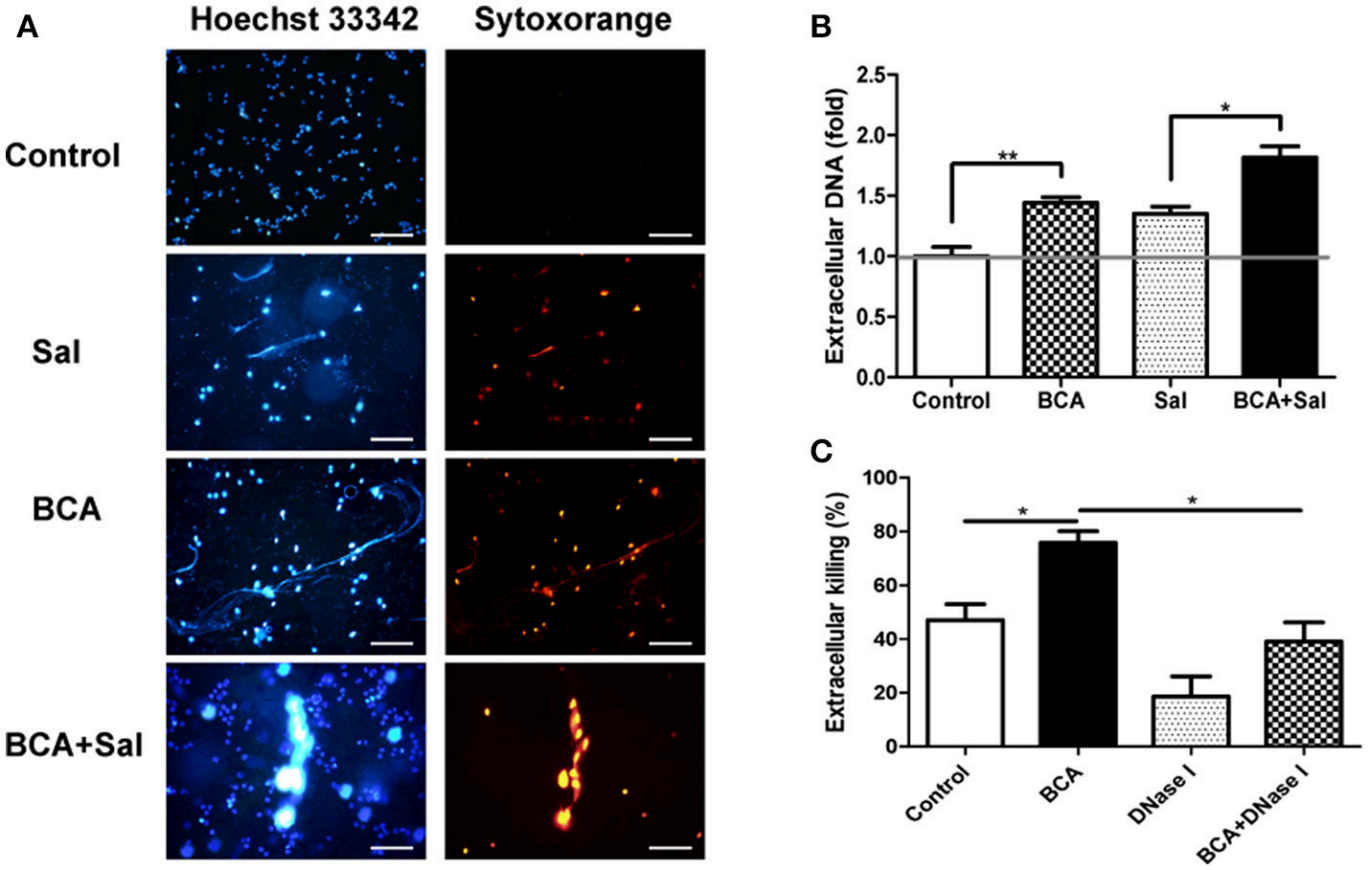
BCA induced MET formation and improved extracellular bacterial killing. **(A,B)** BCA induced Raw264.7 cells to release eDNA. Raw264.7 cells were incubated with serum-free and antibiotic-free medium and treated with 4 μg/ml BCA for 2 h. Then, the cells were infected with *Salmonella* at MOI = 10:1 for 2 h. **(A)** The cells were stained with Hoechst 33342 and Sytox orange and observed by fluorescence microscopy. **(B)** The cells were stained with 1 μM Sytox green for 10 min and detected with a plate reader. ^*^P < 0.05; ^**^P < 0.01. **(C)** Raw264.7 cells pretreated with 10 μg/ml cytochalasin D for 20 min were treated with 4 μg/ml BCA and/or 40 U/ml DNase I and infected with *Salmonella* at MOI = 10:1 for 2 h. The supernatants were collected for CFU quantification and the calculation of the extracellular bacterial killing rate. ^*^P < 0.05. The data are representative of three experiments with similar results.

To investigate whether BCA could induce MET formation and its effect on the extracellular bacterial killing of *Salmonella*, BCA-treated cells were stained with Hoechst 33342 and Sytox Orange and observed with fluorescence microscopy. BCA treatment induced MET formation and significantly increased the MET production of *Salmonella-*infected Raw264.7 cells (Figure 4A); the results were confirmed by the quantification of eDNA (a major component of ETs) with a spectrofluorophotometer (Figure 4B). Furthermore, a phagocytosis inhibition assay showed that BCA significantly increased the amount of extracellular *Salmonella* killed by Raw264.7 cells (*P* < 0.05), while DNase I treatment abolished this effect of BCA (*P* < 0.05) (Figure 4C). Taken together, these results indicate that BCA could kill extracellular *Salmonella* through inducing MET formation.

### AMPK/ULK1/mTOR mediated met formation induced by BCA without ROS involvement

**Figure 5:**
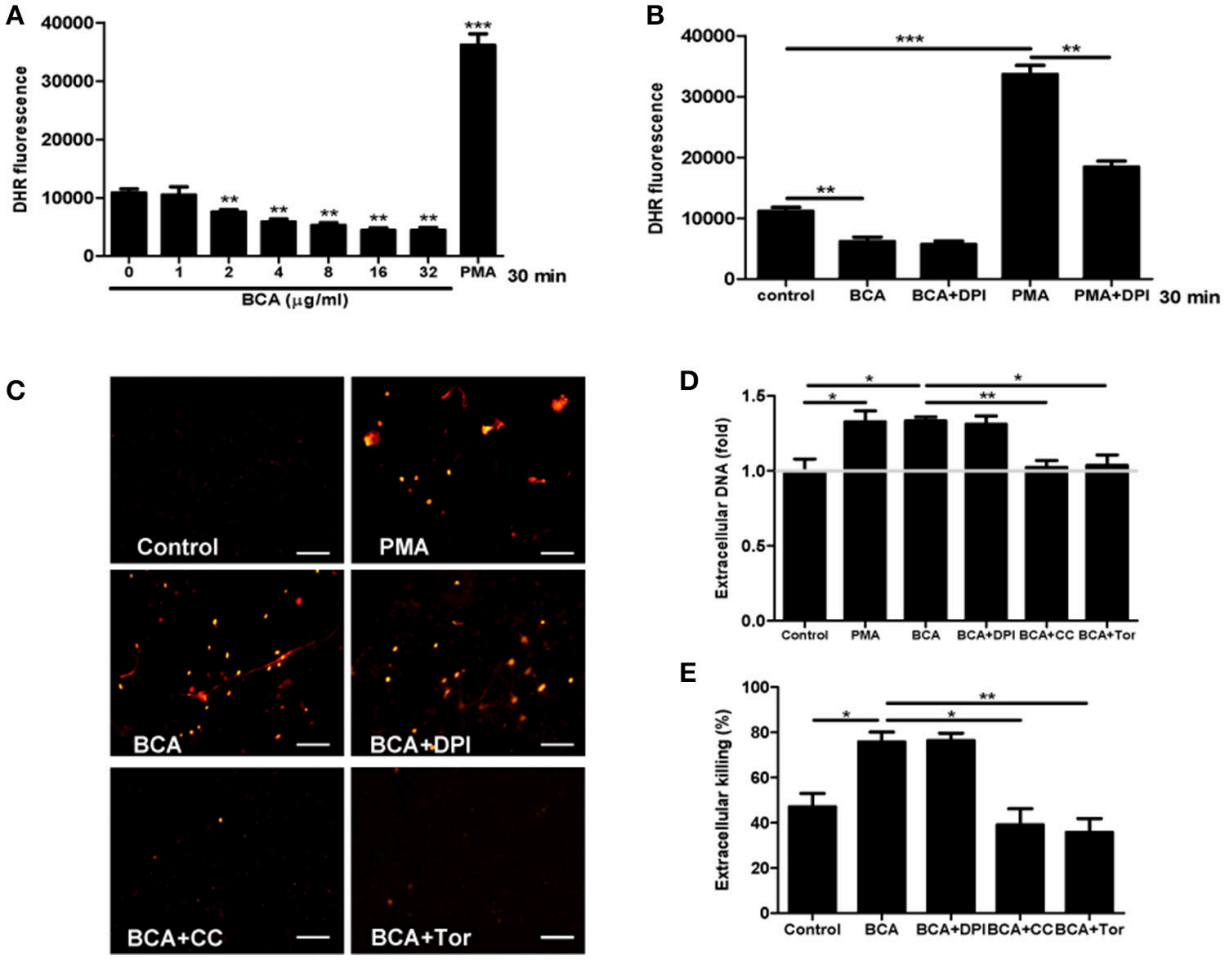
BCA induced AMPK-dependent MET formation without ROS involvement. **(A,B)** Raw264.7 cells were treated with BCA at different concentrations for 30 min or 4 μg/ml for 30 min with or without 50 μM DPI. Then, the cells were incubated with dihydrorhodamine (DHR) 123 for 20 min without light. The plates were examined with a spectrofluorophotometer. ^*^P < 0.05, ^**^P < 0.01, ^***^P < 0.001 compared with the control group. The gray horizontal line represents 1. **(C,D)** Raw264.7 cells that were pretreated with 50 μM DPI or 5 mM compound C or 2 μM Torin 1 for 1 h were treated with 4 μg/ml BCA for 2 h. **(C)** The cells were stained with Sytox orange and observed by fluorescence microscopy. **(D)** The cells were stained with Sytox green and then the results were obtained with a spectrofluorophotometer. ^*^P < 0.05, ^**^P < 0.01. The gray horizontal line represents 1. **(E)** Supernatants were collected for CFU quantification and the calculation of the extracellular bacterial killing rate. ^*^P < 0.05. The data are representative of three experiments with similar results.

To investigate whether ROS takes part in ET formation induced by BCA in MΦ, we quantified the levels of cytosolic ROS and eDNA in BCA-treated Raw264.7 cells with or without DPI (a NADPH inhibitor) (Douda et al., 2015). Phorbol 12-myristate 13-acetate (PMA), a ROS inducer, was used as a positive control. The results showed that BCA inhibited cytosolic ROS production in a dose-dependent manner and 2 μg/ml was the minimal concentration of BCA, which inhibited ROS formation (Figures 5A,B). DPI treatment had no effect on the release of eDNA induced by BCA and the extracellular *Salmonella* killing, while both compound C (an AMPK inhibitor) and Torin 1 (a mTORC1/2 inhibitor) inhibited the release of eDNA induced by BCA and decreased the extracellular bacterial killing (Figures 5C–E). These results indicate that it is AMPK/ULK1/mTOR mediate the MET formation and MET-related extracellular bacterial killing of *Salmonella* induced by BCA, but not ROS.

### BCA enhanced intra- and extra-cellular bacterial killing in the *salmonella*-infected mouse model *in vivo*

**Figure 6:**
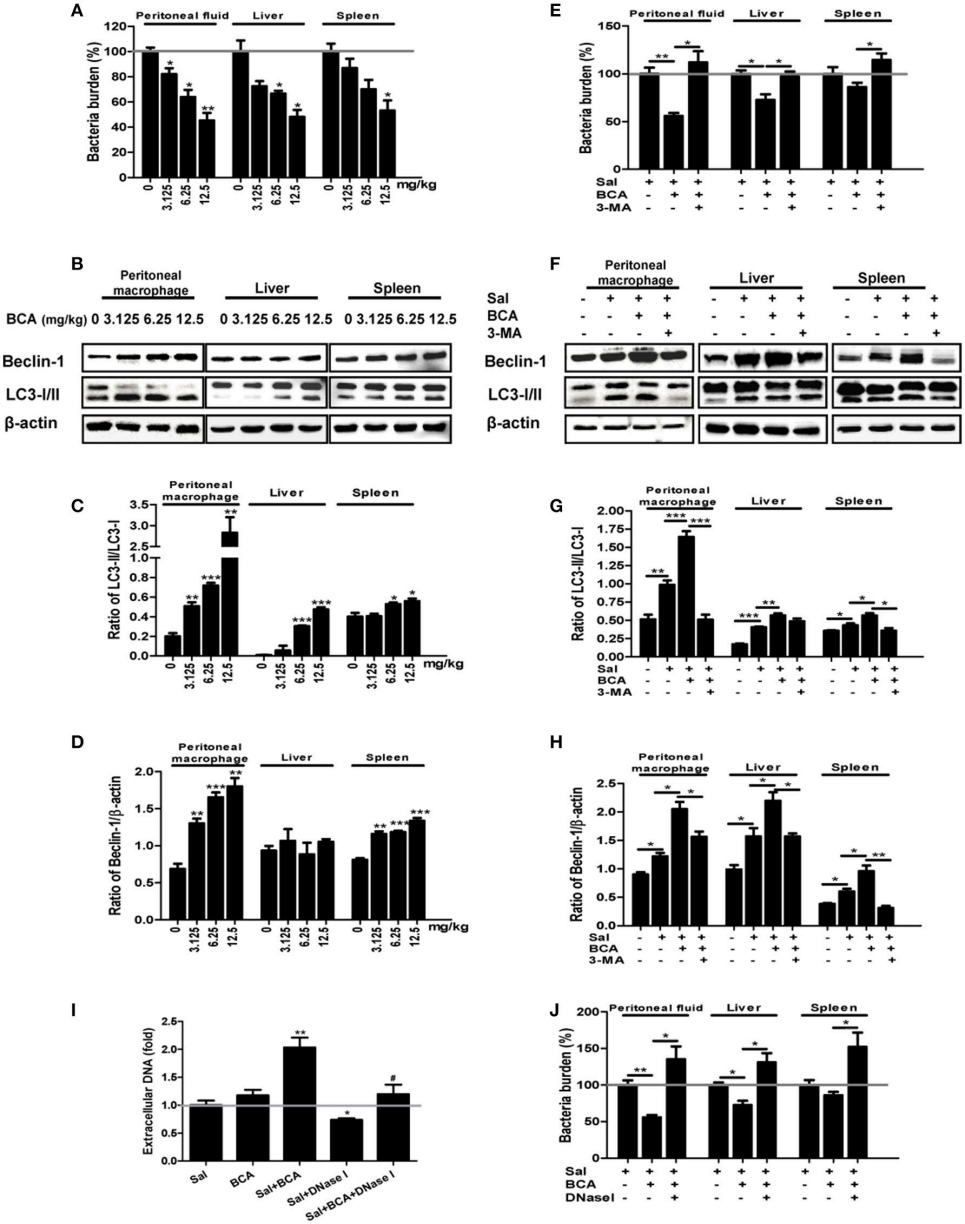
*In vivo* antibacterial activity of BCA. **(A–D)** Mice were infected by intragastric administration of an overnight culture of *Salmonella* (10^5^ bacteria in 0.1 ml PBS) through a gavage tube and then treated with BCA at different doses (3.125–12.5 mg/kg) intragastrically by gavage daily (10 mice for each group). On the 5th day p.i., the mice were sacrificed and the samples were collected. **(A)** The bacteria burdens in the same amount of peritoneal fluid, liver tissue and spleen tissue were quantified. ^*^P < 0.05, ^**^P < 0.01. **(B)** Proteins from peritoneal MΦ, liver tissue and spleen tissue were extracted. **(A)** The expression of LC3, Beclin-1 was detected by western blotting. **(C,D)** The ratios of LC3-II/LC3-I and the ratio of Beclin-1/β-actin were calculated. ^*^P < 0.05, ^**^P < 0.01, ^***^P < 0.001, compared with the control group. **(E–H)** We treated the *Salmonella*-infected mice with 6.25 mg/kg BCA intragastrically by gavage or BCA intragastrically and 24 mg/kg 3-MA by intraperitoneal injection daily for 4 days. On the fifth day after infection, the mice were sacrificed and samples were collected. **(E)** The bacterial burdens in the same amount of peritoneal fluid, liver tissue and spleen tissue were quantified. ^*^P < 0.05, ^**^P < 0.01. **(F)** Proteins were extracted from peritoneal MΦ, liver and spleen tissue. **(E)** The expression of LC3 and Beclin-1 was detected by western blotting. **(G,H)** The ratio of LC3-II/LC3-I and the ratio of Beclin-1/β-actin were calculated. ^*^P < 0.05, ^**^P < 0.01, ^***^P < 0.001. **(I,J)** Mice were infected by the intragastric administration of an overnight culture of *Salmonella* (10^5^ bacteria in 0.1 ml PBS) and then treated with 6.25 mg/kg BCA intragastrically by gavage with or without 100 U DNase I treatment by intraperitoneal injection for 4 days. Peritoneal fluid was stained with Sytox green, and the results were obtained subsequently with a spectrofluorophotometer. ^*^P < 0.05, ^**^P < 0.01, compared with the Salmonella-infected group; ^#^*P* < 0.05, compared with the BCA-treated infected group. The gray horizontal line represents 1. **(J)** The bacteria burdens in the same amount peritoneal fluid, liver tissue and spleen tissue on the fifth day were quantified. ^*^P < 0.05, ^**^P < 0.01.

To assay the effect of enhancement of intra- and extracellular bacterial killing against *Salmonella* by BCA, we conducted the experiment in a *Salmonella*-infected mouse model. BCA treatment markedly reduced the intracellular bacteria counts in a dose dependent manner in the peritoneal lavage fluid and reduced the bacterial burden in liver and spleen tissue (*p* < 0.01, Figure 6A). Treatment with 3-MA (an autophagy inhibitor) reversed the effect of BCA (Figure 6E). Moreover, BCA treatment increased Beclin-1 expression and the LC3-II/LC3-I and Beclin-1/β-actin ratio in peritoneal MΦ, liver, and spleen tissue in a dose dependent manner (Figures 6B–D). Treatment with 3-MA reversed this trend (Figures 6F–H). These data demonstrate that BCA promotes *Salmonella* clearance via autophagy *in vivo*. Next, to determine if BCA improves extracellular bacterial killing through enhancing MET formation *in vivo*, we treated *Salmonella* infected mice with 100 U DNase I (a reagent proven to degrade extracellular traps) by intraperitoneal injection daily (Von Bruhl et al., 2012). BCA treatment caused an increase of eDNA production in peritoneal lavage fluid *ex vivo*, and the treatment of DNase I abrogated the increase (Figure 6I) and increased the bacterial burden in peritoneal lavage fluid, liver and spleen tissue (Figure 6J). This demonstrated that BCA promoted extracellular *Salmonella* clearance via the induction of MET formation *in vivo*. BCA enhanced bactericidal extra- and intra-cellular activity in a *Salmonella* infected mouse model *in vivo*.

### BCA reversed *salmonella* SPI-1-dependent MΦ M2 polarization *in vitro* and *in vivo*

**Figure 7:**
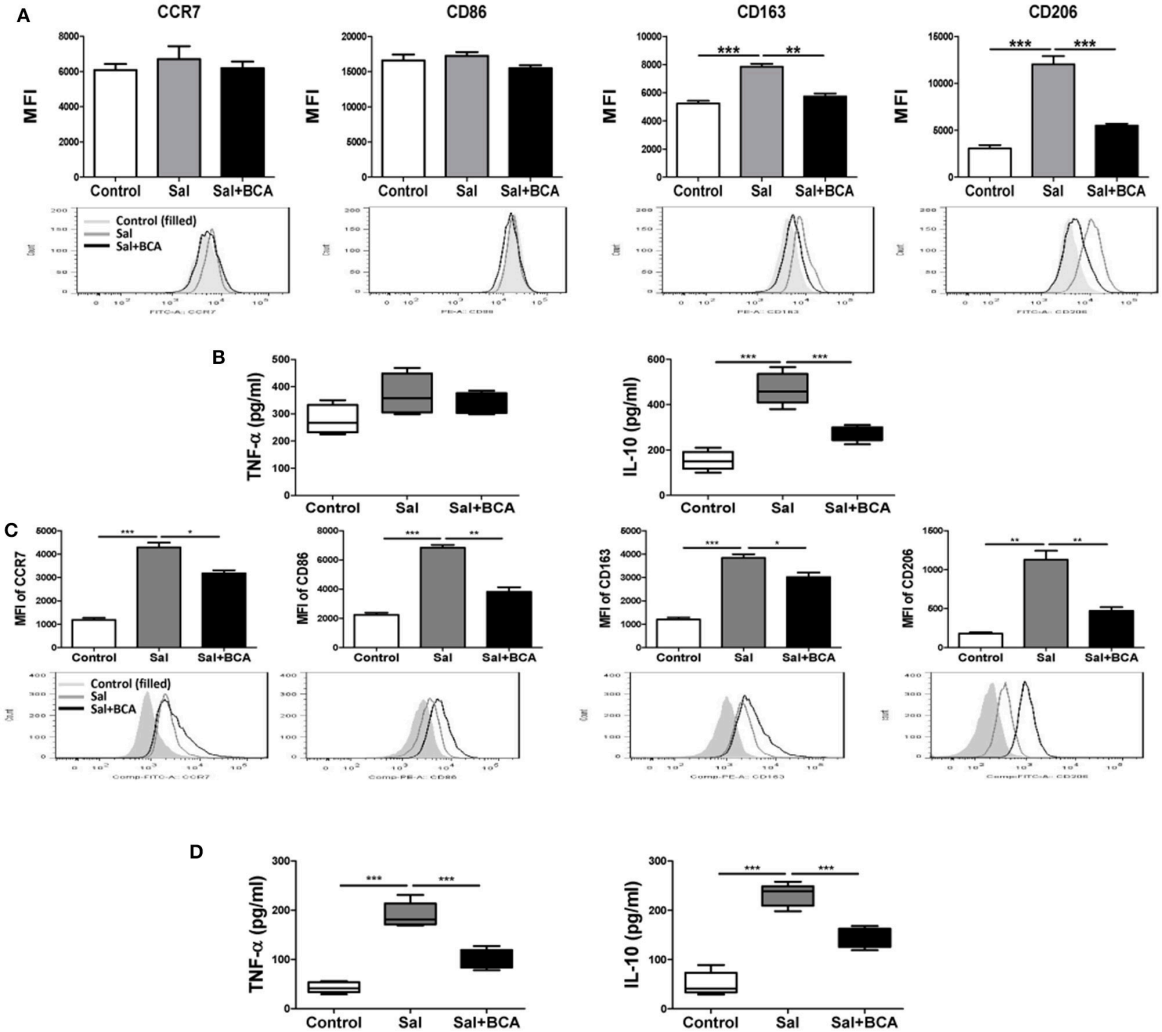
BCA regulate MΦ polarization *in vitro* and *in vivo*. **(A,B)** Raw246.7 cells were treated with 4 μg/ml BCA for 2 h. Then, the cells were infected with *Salmonella* at MOI = 10:1 for 2 h. **(A)** The expression of surface markers (CCR7, CD86, CD163, and CD206) was determined by flow cytometry. The results are presented as MFI. **(B)** TNF-α and IL-10 in the supernatants were detected by ELISA. ^**^P < 0.01, ^***^P < 0.001. The data are representative of three experiments with similar results. **(C,D)** Mice were infected by intragastric administration of an overnight culture of *Salmonella* (10^5^ bacteria in 0.1 ml PBS) through a gavage tube and then treated with 6.25 mg/kg BCA intragastrically by gavage daily (5 mice for each group). On the 5th day p.i., the mice were sacrificed and the peritoneal fluid was collected. **(C)** The expression of surface markers (CCR7, CD86, CD163, and CD206) in mouse peritoneal MΦs were determined by flow cytometry The results are presented as MFI. **(D)** TNF-α and IL-10 in ascitic fluid were detected by ELISA. ^*^P < 0.05, ^**^P < 0.01, ^***^P < 0.001.

To investigate the effect of BCA on MΦ polarization under the condition of *Salmonella*-infection, we detected the markers of M1/M2 phenotypes and cytokines of polarization in Raw246.7 cells *in vitro*. The results showed that the expression levels of M2 MΦ markers CD163 and CD206 increased significantly after infection in Raw246.7 cells, while BCA could reverse this trend partially (Figure 7A). *Salmonella*-infection increased significantly the secretion levels of M2 MΦ marker IL-10 in Raw246.7 cells. Moreover, BCA treatment could down-regulate significantly the levels of IL-10 in the infected macropahges notably (Figure 7B). However, *Salmonella*-infection had no effect on M1 MΦ markers CCR7 and CD86 expression and TNF-α secretion (Figures 7A,B). The previous studies has proved that *Salmonella* induce MΦ polarization to M2 phenotype dependent on SPI-1 gene expression (Kyrova et al., 2012). We tested the SPI-1 gene expression *in vitro* and *in vivo*. Our results showed BCA down-regulated the expression of *hilA* and *hilD* (SPI-1 major genes) significantly (Figures S2A,C–E) and inhibited secretion of SPI-1-encoded protein SipA, SipB and SipC significantly in a dose-dependent manner (Figure S2B). The results indicated that BCA could reverse the M2 phenotype polarization of the *Salmonella*-infected MΦ through down-regulation of SPI-1 expression.

To further test the influence of BCA on Salmonella-infected MΦ plarization *in vivo*, we detected the distinct M1/M2 markers in mouse peritoneal MΦs and the cytokines level in the mouse ascite in the Salmonella-infected mouse model. In mouse peritoneal MΦs, Salmonella infection up-regulated the expression of both of M1 MΦ markers (CCR7 and CD86), M2 MΦ marker (CD163 and CD206) and induced the secretion of M1 MΦ marker TNF-α and M2 MΦ marker IL-10 in ascitic fluid significantly (Figures 7C,D). We found BCA treatment could downregulate the expression of M1 MΦ markers (CCR7 and CD86) and M2 MΦ markers (CD163 and CD206) and decrease the secretion of M1 MΦ marker TNF-α and M2 MΦ marker IL-10 in the Salmonella-infected mouse ascitic fluid significantly (Figures 7C,D). These results indicate that BCA could regulate the bacteria-infected MΦ polarization and could alleviate both inflammatory response and anti-inflammatory response in the case of bacterial infection *in vivo*.

## Discussion

Autophagy has been demonstrated to play an important role in pathogen infection in MΦ and in epithelial cells. Host cells can use autophagy to remove pathogens such as *Mycobacterium tuberculosis, Salmonella*, and *Francisella tularensis* (Chiu et al., 2009; Thurston et al., 2009). Some bacteria such as *Staphylococcus aureus* can escape from killing in host cells with the help of autophagic response (Schnaith et al., 2007). Several agents such as isoniazid, pyrazinamide (Kim et al., 2012), nitazoxanide (Lam et al., 2012), anticonvulsant carbamazepine (Schiebler et al., 2015), and small molecule AR-12 (Chiu et al., 2009) have been suggested to have the ability to stimulate autophagy and inhibit the intracellular proliferation of *M. tuberculosis* and *F. tularensis* (Chiu et al., 2009). In present study, we demonstrated that BCA can induce the complete autophagic response in epithelial cells (HeLa) or MΦ (THP-1 and Raw264.7).

Previous reports of autophagic inducers focused only on their effects on LC3 expression and other possible pathways. In this study, for the first time, we found that BCA could, in addition to the induction of autophagy, restrict the proliferation of invading *Salmonella* by upregulating the expression of the ubiquitinated adapter proteins LRSAM1, NDP52 and p62 and reinforcing the recognition of intracellular bacteria. In addition, BCA treatment increased the colocalization of LAMP1, LysoTracker, LC3 puncta and intracellular bacteria. LAMP1 is a marker of late endosomes/lysosomes that colocalizes with LC3 during autolysosome maturation. LysoTracker is a weak lysosomotropic base that accumulates and fluoresces within acidic vesicles. A gentamicin protection assay verified that BCA improved the killing of intracellular Salmonella through autophagy in HeLa and THP-1 cells. Interestingly, BCA did not show any antibacterial activity *in vitro* against the Salmonella strain used here when tested at concentrations up to 512 μg/mL. Therefore the concentrations of BCA tested were below MIC, demonstrating that the bactericidal intracellular activity of BCA due to an immunomodulating effect rather than the generalized growth inhibition of bacteria. Thus, BCA acts as an enhancer of the host's immune defense and increases the killing of invading Salmonella by autophagy.

We identified the pathways involved in BCA induced autophagy. BCA treatment increased the phosphorylation of AMPK, and Ulk1 Ser 317 and Ser 757, which are phosphorylation sites of AMPK and mTOR, respectively. BCA treatment decreased the phosphorylation of mTOR, 70S6 and 4E-BP1; these results demonstrated that BCA induces autophagy via the AMPK/ULK1/mTOR pathway. AMPK is believed to activate autophagy by inhibiting mTOR complex-1 (Zoncu et al., 2011).

Although *Salmonellae* are facultative intracellular bacteria, MacLennan CA *et al*. have identified that the high fatality rate among young African children is associated with extracellular bacterial growth (Maclennan et al., 2008). Furthermore, it was also indicated that the extracellular killing mechanism was critical to control *Salmonella* in a murine infection model (Siggins et al., 2011). *Salmonella* was reported to induce neutrophil ET formation (Brinkmann et al., 2004). Here, for the first time, we showed that *Salmonella* induced MET formation. To our knowledge, only statins have been demonstrated to be enhancers of bacterial killing by ETs; however, statins inhibit 3-hydroxy 3-methylglutaryl coenzyme A (HMG-CoA) reductase, the rate-limiting enzyme in cholesterol biosynthesis (Chow et al., 2010). This observation suggests that statins may be not suitable for use as enhancers of bacterial killing except in patients that have hypercholesterolemia. Our results showed that BCA enhanced the formation of METs and promotes extracellular *Salmonella* killing *in vitro* and *in vivo*. As a common plant product, BCA is generally considered to be innocuous (Zoncu et al., 2011); our results showed that there was no toxicity at concentrations up to 100 μM in HeLa, RAW 264.7, and THP-1 cell lines, which is consistent with a previous study (Kole et al., 2011).

As the important part of innate immune system and the role of MΦs in immune surveillance and immune regulation, the polarizaiton of MΦs are studied widely. Peripheral monocytes tend to polarize to different subtypes of MΦs according to the tissue microenvironment. The balance of M1/M2 MΦs determines the tissue inflammation and tissue damage (Dey et al., 2014). Different pathogen infections have different effects on the polarization of MΦs and most of them improve MΦs toward M1 phenotype (Khan et al., 2015; Dai et al., 2017; Farias et al., 2017; Hou et al., 2017). *Salmonella* has been proved to induce MΦ polarization toward M2 phenotype depending on SPI-1 (Kyrova et al., 2012), our results *in vitro* were similar to the previous reports. We also demonstrated that BCA could inhibit the polarization to M2 MΦ induced by *Salmonella* infection by down-regulation of SPI-1 genes expression. Noteworthily, we found both expressions of M1 MΦ markers (CCR7 and CD86) and M2 MΦ markers (CD163 and CD206) increased significantly in the peritoneal MΦs of *Salmonella*-infected mice and both levels of TNF-α and IL-10 increased significantly in the infected mice's ascitic fluid, we speculated that there was an imbalance of MΦ M1/M2 polarization associated with inflammatory conditions of *Salmonella* infection, this phenomenon was similar to the results of recent report that *Salmonella* infection could induce both IL-6 (pro-inflammatory) and IL-10 (anti-inflammatory) release in mice (Voinnet, 2011). Thus, our results demonstrated that BCA could deregulate both inflammatory response and anti-inflammatory response in the condition of bacteria infection through regulation of MΦ polarization *in vivo*.

ROS have been reported to mediate ET formation, such as PMA-induced neutrophil ETs (Davidson et al., 2015). In this study, our data showed that BCA inhibited cytosolic ROS release in a dose- and time-dependent manner, this suggested that BCA induced a ROS-independent MET formation, however, our result proved that it was mediated by AMPK/ULK1/mTOR pathway.

In summary, this study demonstrated BCA induced AMPK/ULK1/mTOR-mediated autophagy and METs, which enhanced the defense against *Salmonella* infection *in vitro* and *in vivo*. In the meantime, BCA inhibits both inflammatory response and anti-inflammatory response when the body was infected by bacteria. These findings provide basic data about the control of infections by the enhancement of the host's immune defenses. The use of BCA may represent a new strategy to overcome the desperate scarcity of new therapeutic approaches.

## Author contributions

XZ: data acquisition, data analysis, data interpretation; XT: revising of the manuscript; NG: data acquisition, data analysis; YA: data acquisition, data analysis, data interpretation, writing of the manuscript; XC: data acquisition; CS: data analysis, data interpretation; CW: data analysis, data interpretation; YL, data analysis, data interpretation; SL: revising of the manuscript; HX: revising of the manuscript; ML: reagents, materials, analysis tools provider; YW: reagents, materials, analysis tools provider, the experiments designer; LY: reagents, materials, analysis tools provider, the experiments designer.

### Conflict of interest statement

The authors declare that the research was conducted in the absence of any commercial or financial relationships that could be construed as a potential conflict of interest.
