# Supplementary material for: Biochanin a Enhances the Defense Against Salmonella enterica Infection Through AMPK/ULK1/mTOR-Mediated Autophagy and Extracellular Traps and Reversing SPI-1-Dependent Macrophage (MΦ) M2 Polarization
Source: Front Cell Infect Microbiol. 2018 Sep 11;8:318. doi: 10.3389/fcimb.2018.00318 (PMC6142880; doi:10.3389/fcimb.2018.00318)
Supplement: Supplementary file 1 [file Image_1.PDF]

Fig. S1

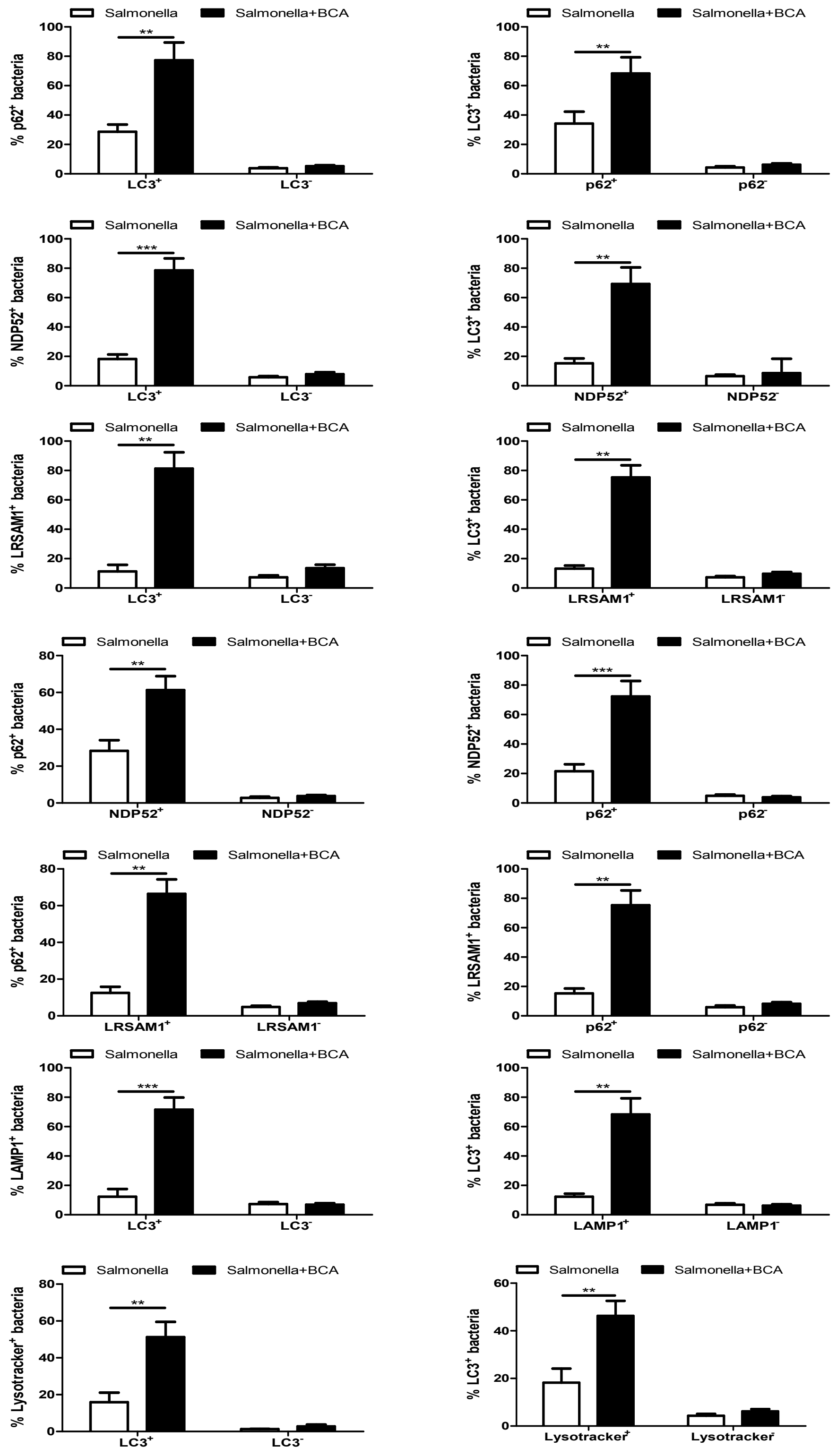

Fig. S1. HeLa cells transfected with pEGFP-LC3 were pretreated with 4 μg/ml BCA for 2 hours and the cells were infected by *Salmonella* for 2 h (MOI=10:1). The cells were coimmunostained with antibodies to NDP52，LRSAM1 and p62 and Hoechst 33342 and analyzed for colocalization of the adaptor proteins and intracellular bacteria or coimmunostained with Lysotracker and Hoechst 33342 and analyzed for colocalization of LAMP1, Lysotracker, LC3 puncta and intracellular bacteria, and were observed using confocal microscopy. The number of intracellular bacteria in 50 cells from each group were counted. ** $P < 0.01$; *** $P < 0.001$. The data are representative of three experiments with similar results.
