# Supplementary material for: Biochanin a Enhances the Defense Against Salmonella enterica Infection Through AMPK/ULK1/mTOR-Mediated Autophagy and Extracellular Traps and Reversing SPI-1-Dependent Macrophage (MΦ) M2 Polarization
Source: Front Cell Infect Microbiol. 2018 Sep 11;8:318. doi: 10.3389/fcimb.2018.00318 (PMC6142880; doi:10.3389/fcimb.2018.00318)
Supplement: Supplementary file 2 [file Image_2.PDF]

Fig. S2

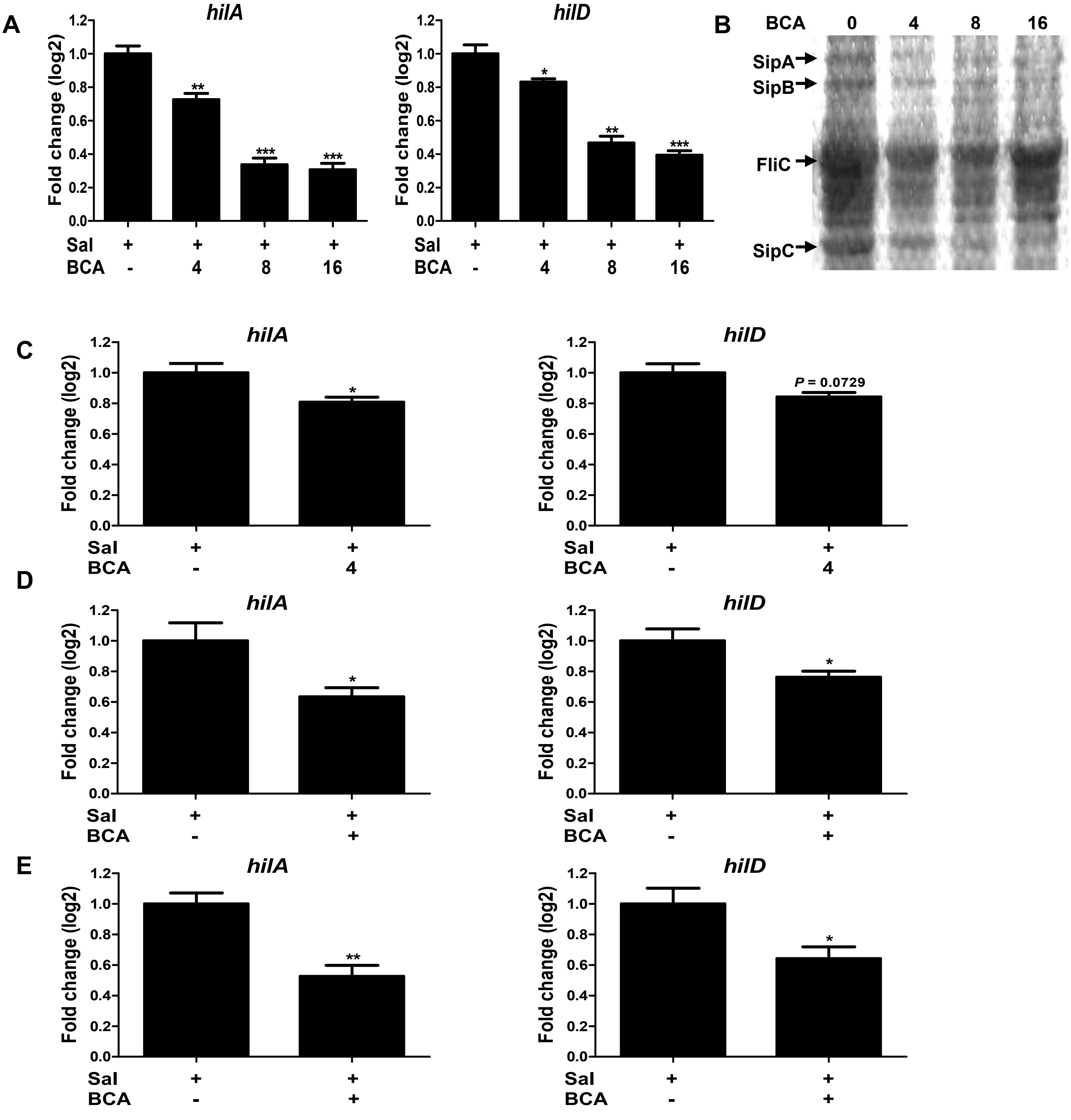

Fig. S2. BCA inhibits the expression of *Salmonella* pathogenicity islands 1 (SPI-1). (A) and (B) *Salmonella* were cultured in LB medium with or without BCA treatment (4 - 16 μg/ml). (A) The expression levels of *hil A* and *hil D* were detection by q-RT-PCR. (B) SDS-PAGE gel pattern of secreted proteins (SPI-1) from *Salmonella* grown in LB medium with or without BCA treatment (4 - 16 μg/ml). (C) THP-1 cells were pretreated with 4 μg/ml BCA for 2 hours and then infected with *Salmonella* for 6 h (MOI = 10:1). The expression levels of *hil A* and *hil D* were detection by q-RT-PCR. (D) and (E) *Salmonella*-infected mice were treated with 6.25 mg/kg BCA intragastrically by gavage daily. On the 5th day p.i., the mice were sacrificed and the expression levels of *hil A* and *hil D* were detection by q-RT-PCR. * $P < 0.05$; ** $P < 0.01$; *** $P < 0.001$. The data are representative of three experiments with similar results.
